# Inhibitory Activities of* Stauntonia hexaphylla* Leaf Constituents on Rat Lens Aldose Reductase and Formation of Advanced Glycation End Products and Antioxidant

**DOI:** 10.1155/2017/4273257

**Published:** 2017-02-23

**Authors:** Seung Hwan Hwang, Shin Hwa Kwon, Set Byeol Kim, Soon Sung Lim

**Affiliations:** ^1^Department of Food Science and Nutrition, Hallym University, 1 Hallymdeahak-gil, Chuncheon 24252, Republic of Korea; ^2^Institute of Natural Medicine, Hallym University, 1 Hallymdeahak-gil, Chuncheon 24252, Republic of Korea; ^3^Institute of Korean Nutrition, Hallym University, 1 Hallymdeahak-gil, Chuncheon 24252, Republic of Korea

## Abstract

*Stauntonia hexaphylla* (Thunb.) Decne. (Lardizabalaceae) leaves (SHL) have been used traditionally as analgesics, sedatives, diuretics, and so on, in China. To date, no data have been reported on the inhibitory effect of SHL and its constituents on rat lens aldose reductase (RLAR) and advanced glycation end products (AGEs). Therefore, the inhibitory effect of compounds isolated from SHL extract on RLAR and AGEs was investigated to evaluate potential treatments of diabetic complications. The ethyl acetate (EtOAC) fraction of SHL extract showed strong inhibitory activity on RLAR and AGEs; therefore, EtOAc fraction (3.0 g) was subjected to Sephadex LH-20 column chromatography, for further fractionation, with 100% MeOH solvent system to investigate its effect on RLAR and AGEs. Phytochemical investigation of SHL led to the isolation of seven compounds. Among the isolated compounds, chlorogenic acid, calceolarioside B, luteolin-3′-*O*-*β*-D-glucopyranoside, quercetin-3-*O*-*β*-D-glucopyranoside, and luteolin-7-*O*-*β*-D-glucopyranoside exhibited significant inhibitory activity against RLAR with IC_50_ in the range of 7.34–23.99 *μ*M. In addition, 3-(3,4-dihydroxyphenyl) propionic acid, neochlorogenic acid, and luteolin-3′-*O*-*β*-D-glucopyranoside exhibited the most potent inhibitory activity against formation of AGEs, with an IC_50_ value of 115.07–184.06 *μ*M, compared to the positive control aminoguanidine (820.44 *μ*M). Based on these findings, SHL dietary supplements could be considered for the prevention and/or treatment of diabetes complication.

## 1. Introduction

Diabetic complications including neuropathy, nephropathy, cataracts, and retinopathy are considered to result from the accumulation of sorbitol, which is obtained from reduction of glucose by the catalytic activity of aldose reductase (AR, EC 1.1.1.21) in the polyol pathway. Sorbitol is subsequently metabolized to fructose by sorbitol dehydrogenase [1]. The increased polyol pathway flux leads to the accumulation of sorbitol in the lens fiber, causing an influx of water, generation of osmotic stress, and formation of cataracts which is the leading cause of blindness worldwide in patients with diabetes. Therefore, AR inhibition represents a key point for the prevention and attenuation of long-term diabetic complications [2].

Advanced glycation end products (AGEs) are proteins or lipids that become glycated after exposure to sugars. AGEs are nonenzymatic adducts of protein, lipids, and nucleic acids, formed in a time-dependent manner in a prooxidant environment, especially when the target molecules are slowly metabolized and the levels of aldoses are high [3]. In particular, during hyperglycemia, body proteins undergo increased glycation, where glucose reacts nonenzymatically with protein amino groups to form a labile Schiff base that rearranges to a stable Amadori product. The formation and accumulation of AGEs in many different cell types affect extracellular and intracellular structure and functions by inducing oxidative stress [4].

*Stauntonia hexaphylla* (Thunb.) Decne. (Lardizabalaceae) is widely distributed as thickets in lowlands and foothills of warmer regions of Korea, Japan, and China.* S. hexaphylla* has been traditionally used in China as analgesic, sedative, and diuretic, among other purposes [5]. In recent years, much attention has been paid to* S. hexaphylla* mainly due to its various biological activities, particularly, anti-HIV-1 [6], anti-inflammatory [7], and cytotoxic properties [8]. The known chemical constituents of* S. hexaphylla* include triterpenoids, glucosides, flavonoids, phenylpropanoids, phenolic glucosides, and chlorogenic acid analogues [9].

To date, however, no data are available on the inhibitory effects of the* S. hexaphylla* leaves' (SHL) constituents on rat lens aldose reductase (RLAR) and AGEs. In the present study, we investigated the inhibitory effect of compounds isolated from SHL on RLAR and AGEs to evaluate their potential to treat diabetic complications.

## 2. Materials and Methods

### 2.1. Chemicals and Reagents

DL-Glyceraldehyde, reduced form of nicotinamide adenine dinucleotide phosphate (NADPH), bovine serum albumin, methylglyoxal, 2,2-diphenyl-1-picrylhydrazyl (DPPH), aminoguanidine, L-ascorbic acid, and quercetin used in this study were purchased from Sigma (St. Louis, MO, USA). All other chemicals and reagents used were of analytical grade.

### 2.2. Instruments

^1^H and ^13^C NMR spectra and correlation NMR spectra such as COSY, HMBC, and HMQC were obtained from an Avance DPX 400 (or 600) spectrometer (Bruker, Madison, WI, USA). These were obtained at operating frequencies of 400 MHz (or 600) (^1^H) and 100 (or 150) MHz (^13^C) with CD_3_OD, (CD_3_)_2_SO, and TMS were used as internal standards; chemical shifts were reported in *δ* values. The molecular mass was measured using the Voyager DE STR matrix assisted laser desorption/ionization time-of-flight (MALDI-TOF) mass spectrometer (MS, Applied Bio-systems, Foster City, CA, USA), the low resolution electronic impact (EI) MS equipped JMS-700 (Tokyo, Japan). The compounds were dissolved in methanol (MeOH) and mixed with a matrix, *α*-cyano-4-hydroxycinnamic acid. The ratio of the amount of the sample and matrix was 1 : 1 (v/v). The mixture was spotted on a stainless steel plate and dried at room temperature. After the water vaporized, MALDI-TOF analysis was performed with an accelerating voltage of 20 kV. The low resolution MS was operated in the negative-ion mode with ion source at 250°C and EI at 70 eV with direct insertion probe and the mass range in 50–600* m/z*. Fast atom bombardment (FAB) MS was recorded in the negative form using* m*-nitrobenzyl alcohol as matrix in a JEOL JMSAX 505-WA spectrometer (Tokyo, Japan). Column chromatography procedures were performed on Sephadex LH-20 (GE Healthcare, Uppsala, Sweden).

### 2.3. Plant Material and Preparation of Extract

Dried SHL were purchased from Dae-Kwang Herb Medicine Co., Ltd. (Chuncheon, Korea) and the voucher specimen (number 2016-RIC-0321) was deposited at the Regional Innovation Center, Hallym University, Korea. The specimen was authenticated by Emeritus Prof. H. J. Chi, Seoul National University, Korea.

### 2.4. Extraction, Fractionation, and Isolation

**Figure 1:**
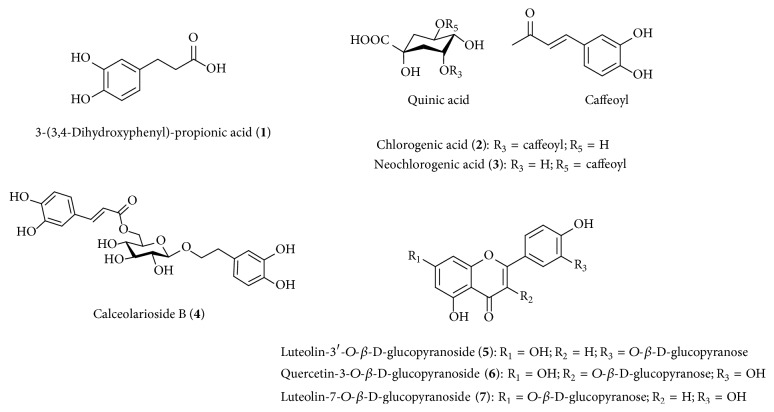
Chemical structures of the compounds isolated from* Stauntonia hexaphylla* leaves.

The dried SHL (1 kg) was extracted with 70% ethanol (2 L × 2 times) for 2 h at room temperature. The combined filtrates were concentrated to dryness in vacuo at 40°C. The dried extract was suspended (160 g) in distilled water and partitioned sequentially with* n*-hexane (Hex, 3.8 g), methylene chloride (CH_2_Cl_2_, 10.02 g), ethyl acetate (EtOAc, 13.54 g),* n*-butanol (*n*-BuOH, 62.29 g), and water residue (water, 60.69 g). The EtOAc fraction showed strong inhibitory effect on RLAR and AGEs (Table 1); therefore, EtOAc fraction (3 g) was chromatographed over a Sephadex LH-20 column using MeOH as the eluent to obtain eight pooled fractions (SHLFs 1–8). SHLFs 1 and 2 were further fractionated using Sephadex LH-20 column chromatography and MeOH-H_2_O (4 : 1, v/v) as the eluent to give compounds** 1** (4.8 mg),** 2** (23.9 mg),** 3** (18.6 mg),** 4** (9.4 mg), and** 5** (10.3 mg). SHLF 6 was subjected to Sephadex LH-20 column chromatography with MeOH-H_2_O (1 : 1, v/v) as the eluent to give compounds** 6** (6.9 mg) and** 7** (8.5 mg) (Figure 1).

### 2.5. Preparation of Aldose Reductase

Crude RLAR was prepared as follows: lenses weighing 250–280 g were removed from Sprague-Dawley rats and frozen at −70°C until use. Noncataractous transparent lenses were pooled and a homogenate was prepared in 0.1 M phosphate buffer saline (pH 6.2). RLAR homogenate was then centrifuged at 9600 ×g for 20 min. After centrifugation in a refrigerated centrifuge, the supernatant was collected and used as the RLAR. All procedures were carried out at 4°C [10].

### 2.6. In Vitro Determination of RLAR Inhibition

RLAR activity was assayed spectrophotometrically by measuring the decrease in the absorption of NADPH at 340 nm over a 4 min period using DL-glyceraldehyde as the substrate. Each 1.0 mL cuvette contained equal units of the enzyme, 0.1 M sodium phosphate buffer (pH 6.2), 0.3 mM NADPH, with or without 10 mM of the substrate, and an inhibitor [11]. The concentration of inhibitors causing 50% inhibition of enzyme activity (IC_50_) was calculated from the least squares regression line of the logarithmic concentrations plotted against the residual activity.

### 2.7. Bovine Serum Albumin-Methylglyoxal Assay on AGEs Formation

Bovine serum albumin (50 mg/mL) was incubated with methylglyoxal (100 mM) in sodium phosphate buffer (0.1 M, pH 7.4) in the presence of various concentrations of the compounds (including a control) at 37°C for 24 h. Then the fluorescent intensity was measured at an excitation wavelength of 355 nm and an emission wavelength of 460 nm with a luminescence spectrometer LS50B (Perkin-Elmer Ltd., Buckinghamshire, England) [4]. The dimethylsulfoxide used as vehicle was found to have no effect on the reaction. All reagents and samples were sterilized by filtration through 0.2 mm membrane filters.

### 2.8. DPPH Assay

The stable free radical, DPPH, was used to determine the free radical scavenging activity of the SHL extracts. Briefly, a 0.32 mM solution of DPPH in methanol was prepared, and then 180 *µ*l of this solution was mixed with 30 *µ*l of each sample (1.0 to 5.0 mg/mL in methanol). After 15 min of incubation in the dark, the decrease in the absorbance of the solution was measured at 570 nm on a microplate reader (EL800 Universal Microplate reader, Bio-Tek instruments, Winooski, VT, USA).

### 2.9. HPLC Analysis of EtOAc Fraction of SHL

The HPLC analysis was performed on an Agilent 1100 series system equipped with a diode-array detector (DAD, Agilent, Sunnyvale, CA, USA) consisting of a vacuum degasser (G1322A), a quaternary pump (G1311A), an autosampler (G1313A), a thermostated column compartment (G1316A), and a DAD (G1315B) system. Separation was achieved on an Eclipse XDB-phenyl column (150 mm × 4.6 mm, 3.5 *μ*m), coupled with a guard column, at 30°C. 10 *µ*l of samples was injected into the system. The samples were eluted with acidified water (0.1% trifluoroacetic acid, A) and methanol (B), at a flow rate of 0.7 mL/min. The optimized gradient chromatographic conditions were as follows: 5–100% B at 0–32 min; 100–5% B at 32–35 min; isocratic 5% B at 35–40 min. The detector monitored the eluent at wavelength 254 nm. Calibration curves were peak area versus concentration for each standard solution. Serially diluted solutions of the seven standard compounds prepared in the range of 1, 10, 25, 50, 75, and 100 *µ*g/mL were injected into the HPLC for quantification. The limit of quantification (LOQ) was determined as the concentration with a signal-to-noise ratio of ten.

## 3. Results

### 3.1. Structural Determination of Isolated Compounds

Seven compounds were separated from EtOAc fraction by Sephadex LH-20 column chromatography. These compounds were identified by comparing ^1^H and ^13^C NMR spectra and correlation NMR spectra such as COSY, HMBC, and HMQC with previously reported data and EI-, FAB-, and MALDI-TOF MS. The seven compounds are compounds** 1** (3-(3,4-dihydroxyphenyl)-propionic acid) [12],** 2** (chlorogenic acid) [10],** 3** (neochlorogenic acid) [13],** 4** (calceolarioside B) [14],** 5** (luteolin-3′-*O*-*β*-D-glucopyranoside) [15],** 6** (quercetin-3-*O*-*β*-D-glucopyranoside) [16], and** 7** (luteolin-7-*O*-*β*-D-glucopyranoside) [17].

*Compound* ***1***. EI-MS* m/z* 183 [M + H]^+^, 165 [M-OH]^+^, 138 [M-COOH]^+^. UV (MeCN, *λ*_max_ nm) 229, 254, 290. ^1^H NMR (400 MHz, CD_3_OD): *δ* 6.87 (1H, d,* J* = 1.84 Hz, H-2), 6.69 (1H, d, *J* = 8.01 Hz, H-5), 6.64 (1H, dd, *J* = 8.01 and 1.84 Hz, H-6), 2.77 (2H, t, *J* = 7.38 Hz, H-7ab), 2.52 (2H, t, *J* = 7.70 Hz, H-8b). ^13^C NMR (100 MHz, CD_3_OD): *δ* 174.40 (C-9), 146.08 (C-3), 144.61 (C-4), 131.40 (C-1), 121.26 (C-6), 117.07 (C-2), 116.36 (C-5), 36.71 (C-8), 29.94 (C-7).

*Compound* ***2***. MALDI-TOF MS* m/z* 377.1275 [M + Na]^+^, 400.1173 [M + 2Na]^+^. UV (MeCN, *λ*_max_ nm) 298, 346. ^1^H NMR (400 MHz, CD_3_OD): *δ* 7.55 (1H, d, *J* = 15.91 Hz, H-7′), 7.04 (1H, d, *J* = 1.80 Hz, H-2′), 6.94 (1H, dd, *J* = 8.21 Hz and *J* = 1.80 Hz, H-6′), 6.77 (1H, d, *J* = 8.23 Hz, H-5′), 6.26 (1H, d, *J* = 15.92 Hz, H-8′), 5.34 (1H, m, H-3), 4.17 (1H, m, H-5), 3.72 (1H, dd, *J* = 8.51 Hz and *J* = 3.03 Hz, H-4), 2.21 (2H, m, H-6), 2.05 (2H, m, H-2). ^13^C NMR (100 MHz, CD_3_OD): *δ* 175.95 (C-7), 167.34 (C-9′), 148.15 (C-4′), 145.68 (C-7′), 145.39 (C-3′), 126.41 (C-1′), 121.59 (C-6′), 115.10 (C-8′), 113.90 (C-5′), 113.82 (C-2′), 74.92 (C-1), 72.24 (C-3), 70.63 (C-4), 70.09 (C-5), 37.59 (C-6), 36.87 (C-2).

*Compound* ***3***. MALDI-TOF MS* m/z* 377.0947 [M + Na]^+^, 400.0845 [M + 2Na]^+^. UV (MeCN, *λ*_max_ nm) 243, 328. ^1^H NMR (600 MHz, CD_3_OD): *δ* 7.58 (1H, d, *J* = 15.93 Hz, H-7′), 7.04 (1H, br s, H-2′), 6.93 (1H, d, *J* = 8.01 Hz, H-6′), 6.77 (1H, d, *J* = 8.03 Hz, H-5′), 6.31 (1H, d, *J* = 15.94 Hz, H-8′), 5.36 (1H, br s, H-5), 4.13 (1H, s, H-3), 3.66 (1H, m, H-4), 2.14 (2H, m, H-6), 1.97 (2H, m, H-2). ^13^C NMR (125 MHz, CD_3_OD): *δ* 177.66 (C-7), 167.65 (C-9′), 148.03 (C-4′), 145.47 (C-7′), 145.37 (C-3′), 126.59 (C-1′), 121.52 (C-6′), 115.10 (C-2′), 114.42 (C-5′), 113.78 (C-8′), 78.93 (C-1), 73.18 (C-5), 71.56 (C-4), 67.20 (C-3), 39.78 (C-6), 35.43 (C-2).

*Compound* ***4***. MALDI-TOF MS* m/z* 501.1398 [M + Na]^+^, 524.1296 [M + 2Na]^+^. UV (MeCN, *λ*_max_ nm) 218, 327. ^1^H NMR (400 MHz, CD_3_OD): *δ* 7.55 (1H, d, *J* = 15.86 Hz, H-7′′), 7.03 (1H, d, *J* = 1.92 Hz, H-2′′), 6.88 (1H, dd, *J* = 8.24 and 1.92 Hz, H-6′′), 6.76 (1H, d, *J* = 8.23 Hz, H-5′′), 6.67 (1H, d, *J* = 1.91 Hz, H-2), 6.63 (1H, d, *J* = 8.12 Hz, H-5), 6.53 (1H, dd, *J* = 8.12 and 1.92 Hz, H-6), 6.28 (1H, d, *J* = 15.87 Hz, H-8′′), 4.49 (1H, dd, *J* = 11.91 and 1.90 Hz, H-6′a), 4.35 (1H, br d, *J* = 5.73 Hz, H-6′b), 4.32 (1H, d, *J* = 8.12 Hz, H-1′), 4.00 (1H, m, H-8a), 3.72 (1H, m, H-8b), 3.55-3.32 (4H, m, H-2′, 3′, 4′ and 5′), 2.77 (2H, m, H-7ab). ^13^C NMR (100 MHz, CD_3_OD): *δ* 169.13 (C-9′′), 149.57 (C-4′′), 147.23 (C-7′′), 146.73 (C-3′′), 146.08 (C-3), 144.61 (C-4), 131.40 (C-1), 127.68 (C-1′′), 123.13 (C-6′′), 121.26 (C-6), 117.07 (C-2), 116.54 (C-5′′), 116.36 (C-5), 115.10 (C-2′′) 114.83 (C-8′′), 104.35 (C-1′), 75.65 (C-2′), 75.38 (C-5′), 72.96 (C-3′), 72, 32 (C-8), 70.37 (C-4′), 64.61 (C-6′), 36.65 (C-7).

*Compound* ***5***. FAB-MS* m/z* 449 [M + H]^+^, 287 [M + H-glucse]^+^. UV (MeCN, *λ*_max_ nm) 265, 296, 331. ^1^H NMR (400 MHz, CD_3_OD): *δ* 7.42 (1H, dd, *J* = 8.17 and 2.12 Hz, H-6′), 7.38 (1H, d, *J* = 2.51 Hz, H-2′), 6.79 (1H, d, *J* = 8.53 Hz, H-5′), 6.62 (1H, d, *J* = 1.87 Hz, H-8), 6.58 (1H, s, H-3), 6.43 (1H, d, *J* = 1.87 Hz, H-6), 5.32 (1H, d, *J* = 7.51 Hz, H-1′′), 4.01–3.53 (6H, m, H-2′′, 3′′, 4′′, 5′′ and 6′′ab). ^13^C NMR (100 MHz, CD_3_OD): *δ* 187.77 (C-4), 169.21 (C-7), 166.39 (C-2), 163.58 (C-5), 159.93 (C-9), 156.87 (C-4′), 153.22 (C-3′), 126.74 (C-1′), 121.77 (C-6′), 118.54 (C-5′), 119.87 (C-2′), 107.94 (C-3), 108.13 (C-10), 102.15 (C-1′′), 98.22 (C-6), 96.92 (C-8), 76.34 (C-5′′), 74.80 (C-3′′), 73.97 (C-2′′), 72.54 (C-4′′), 63.33 (C-6′′).

*Compound* ***6***. FAB-MS* m/z* 465 [M + H]^+^, 303 [M + H-glucse]^+^. UV (MeCN, *λ*_max_ nm) 257, 359. ^1^H-NMR (400 MHz, (CD_3_)_2_SO): *δ* 7.48 (1H, dd, *J* = 8.20 and 1.99 Hz, H-6′), 7.47 (1H, d, *J* = 2.27 Hz, H-2′), 6.78 (1H, d, *J* = 8.19 Hz, H-5′), 6.32 (1H, d, *J* = 1.67 Hz, H-8), 6.13 (1H, d,* J* = 1.68 Hz, H-6), 5.28 (1H, d, *J* = 7.20 Hz, H-1′′), 3.23-3.41 (6H, m, H-2′′, 3′′, 4′′, 5′′ and 6′′ab). ^13^C-NMR (100 MHz, *δ*, (CD_3_)_2_SO): *δ* 177.33 (C-4), 164.06 (C-7), 161.18 (C-5), 156.56 (C-9), 156.38 (C-2), 148.37 (C-4′), 144.71 (C-3′), 133.28 (C-3), 121.54 (C-1′), 121.14 (C-6′), 116.23 (C-5′), 115.18 (C-2′), 103.92 (C-10), 101.16 (C-1′′), 98.64 (C-6), 93.54 (C-8), 76.42 (C-3′′), 75.87 (C-5′′), 74.04 (C-2′′), 70.53 (C-4′′), 66.95 (C-6′′).

*Compound* ***7***. FAB-MS* m/z* 449 [M + H]^+^, 287 [M + H-glucse]^+^. UV (MeCN, *λ*_max_ nm) 256, 266, 348. ^1^H NMR (400 MHz, CD_3_OD): *δ* 7.44 (1H, dd, *J* = 8.43 and 2.02 Hz, H-6′), 7.34 (1H, d, *J* = 1.90 Hz, H-2′), 6.87 (1H, d, *J* = 8.63 Hz, H-5′), 6.68 (1H, d, *J* = 1.82 Hz, H-8), 6.50 (1H, s, H-3), 6.45 (1H, d, *J* = 1.81 Hz, H-6), 5.29 (1H, d, *J* = 7.39 Hz, H-1′′), 4.16–3.42 (6H, m, H-2′′, 3′′, 4′′, 5′′ and 6′′ab). ^13^C NMR (100 MHz, CD_3_OD): *δ* 184.38 (C-4), 166.62 (C-7), 165.24 (C-2), 162.41 (C-5), 159.08 (C-9), 151.42 (C-4′), 147.42 (C-3′), 123.93 (C-1′), 120.73 (C-6′), 117.19 (C-5′), 114.56 (C-2′), 109.54 (C-3), 105.61 (C-10), 104.30 (C-1′′), 97.61 (C-6), 95.40 (C-8), 77.53 (C-5′′), 75.71 (C-3′′), 73.02 (C-2′′), 72.20 (C-4′′), 63.29 (C-6′′).

### 3.2. Inhibitory Activity of Isolated Compounds on RLAR

A 70% ethanol extract of SHL was found to exhibit inhibitory activity against RLAR (34.52 *μ*g/mL), AGEs (422.51 *μ*g/mL), and DPPH (193.19 *μ*g/mL) compared with positive controls quercetin (5.48 *μ*g/mL), aminoguanidine (90.70 *μ*g/mL), and L-ascorbic acid (9.58 *μ*g/mL). The extract was suspended in distilled water and partitioned with with Hex, CH_2_Cl_2_, EtOAc, n-BuOH, and water residue. Among fractions, the EtOAc fraction exhibited potent inhibitory activity against RLAR with IC_50_ value of 6.90 *μ*g/mL. Therefore, this study focused on the isolation of AR inhibitors from this fraction (Table 1). The inhibitory activities of the compounds** 1**–**7** isolated from EtOAc fraction were evaluated on RLAR. Of the tested compounds,** 2**,** 5**,** 6**, and** 7** showed strong inhibitory activities on RLAR with IC_50_ value of 8.35, 7.34, 10.40, and 16.10 *μ*M, respectively (Table 2), which were higher than that of positive control (quercetin, 18.09 *μ*M). In addition, compounds** 3** and** 4** showed inhibitory effect against RLAR with IC_50_ of 72.03 and 23.99 *μ*M, respectively.

### 3.3. Inhibitory Activity of Isolated Compounds on AGEs

The extract and fractions were evaluated for AGEs using a bovine serum albumin-methylglyoxal assay. All the extract and fractions showed significant inhibitory activity with IC_50_ values ranging from 9.80 to 28.44 *μ*g/mL (Table 1). Among the fractions, the EtOAc fraction (50.07 *μ*g/mL) was found to exhibit a similar activity to the positive control, a known AGEs inhibitor (aminoguanidine, 90.70 *μ*g/mL). Among isolated compounds, AGEs activity results showed that compound** 3** had the highest inhibitory effect against AGEs formation among isolated compounds with IC_50_ value of 115.07 *μ*M; also, compounds** 1** and** 5** had IC_50_ values of 184.06 and 117.80 *μ*M, respectively (Table 3). However, other compounds had no inhibitory effect even at the same concentration when compared to aminoguanidine (820.44 *μ*M).

### 3.4. Antioxidant Activity of Isolated Compounds on ABTS^+^

The antioxidant activity of the fractions and the constituents was evaluated in vitro by examining the DPPH radical scavenging activity. As shown in Table 1, the EtOAc fraction of SHL exhibited potent inhibitory activity against DPPH (IC_50_ value: 63.00 *μ*g/mL) when compared with that of the positive control (L-ascorbic acid, 9.58 *μ*g/mL). Among the isolated compounds from the EtOAc fraction, most compounds were found to have inhibitory activities with IC_50_ in the range of 94.60–162.60 *μ*M (Table 4). Of the tested, compounds** 6** and** 10** showed strong inhibitory activity with IC_50_ values of 18.42 and 88.14 *μ*M, respectively. However, compounds** 3** and** 7** had no an inhibitory effect even at the same concentration when compared to other compounds.

### 3.5. Quantification of Isolated Compounds from SHL EtOAc Fraction by HPLC

**Figure 2:**
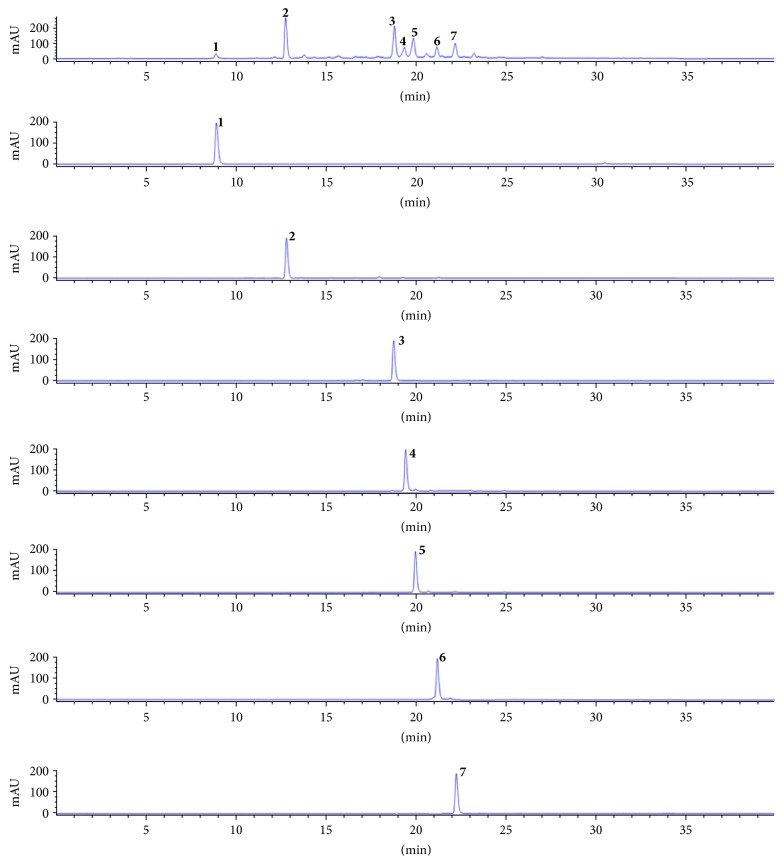
HPLC profile of seven compounds isolated from EtOAc fraction of* Stauntonia hexaphylla*, 70% ethanol extract at 254 nm.

In addition, compounds** 1**,** 2**,** 3**,** 4**,** 5**,** 6**, and** 7** in the EtOAc were analyzed with HPLC-DAD (Figure 2). Based on the established method, these compounds (**1–7**) identified the contents of 3.07, 24.89, 16.80, 4.10, 10.19, 5.14, and 7.77% in SHL extract, respectively. And, the purity of all compounds isolated was high purities: compound** 1** (98.73%),** 2** (96.15%),** 3** (98.83%),** 4** (96.83%),** 5** (96.63%),** 6** (95.13%), and** 7 **(98.36%), respectively.

## 4. Discussion

The inhibitory behavior of SHL constituents and the related structural activity relationship were investigated using the RLAR assay. RLAR-inhibitory potential of SHL constituents depends on the site of quinic acid and/or other residues of the -COOH position in the caffeic acid structure (compound** 2** >** 4** >** 3**). A possible mechanism by which caffeoylquinic acid inhibits RLAR could be related to its structure's action position [11]. In addition, many structural properties of the sugar position in flavonoids that inhibit RLAR have been reported. Increasing the sugar residue at the 3′ position of the B ring in luteolin (aglycone of compound** 5**) increases the inhibitory activities against RLAR; in contrast, increasing the sugar residue at the 7′ positions of the A ring of luteolin decreases the inhibitory activities against RLAR (compound** 5** >** 6** >** 7**) [18]. However, AGEs and DPPH results have no significant relationship between structure and their inhibitory activity.

Hyperglycemia plays an important role in the pathogenesis of diabetic complications associated with vascular and nerve damage by several mechanisms such as increased AR-related polyol pathway flux, increased AGEs formation, and excessive oxidation stress [19]. Among them, cataract formation is the leading cause of blindness worldwide in diabetic patients and, especially, must be considered in patients with uncontrolled diabetes. AR inhibitors have received considerable attention because of the proposed involvement of AR in the pathophysiology of diabetic complications including cataract [20]. In addition, AR-catalyzed formation of sorbitol was also observed in a number of tissues and in diabetes mellitus; increased sorbitol through the polyol pathway does not readily diffuse across cell membranes and the intracellular accumulation of sorbitol has been implicated in the chronic complications of diabetes such as cataract, neuropathy, and retinopathy [21]. These findings suggested that ARIs prevent the conversion of glucose to sorbitol and may have the capacity of preventing and/or treating several diabetic complications [22]. In addition, the formation and accumulation of AGEs in various tissues have been reported to progress at an accelerated rate under hyperglycemic conditions. The formation and accumulation of AGEs will induce oxidative stress and it would have deleterious effects on various cellular functions [23].

Various natural extracts and their constituents have long been used in traditional herbal medicine, particularly in the treatment of diabetes and diabetic complication. In most cases, natural extracts could have less side effects and lower toxicity. Therefore, there is growing interest in using natural products as sources of new drugs [24]. Flavonoid and its derivatives are an interesting chemical group of natural products that are widely distributed in the various plants and most of these compounds are isolated from medicinal plants. A recent study reported that the constituents isolated from* Abeliophyllum distichum* [25],* Zea mays* L. [1], and* Perilla frutescens* [26] showed an inhibitory effect on rat lens AR activity. Polyphenols including rosmarinic acid and caffeic acid ethylene ester isolated from* Prunella vulgaris* L. displayed therapeutic potential in the prevention and treatment of diabetic complications by inhibiting AR and protein glycation [27]. Our results suggest that SHL and its constituents prevent cataractogenesis by inhibiting AR activity. For this reason, the pursuit for new AR inhibitors of natural origin is highly justified.

## 5. Conclusion

In summary, seven compounds were isolated from the EtOAc fraction of the SHL and the inhibitory effect of the compounds was evaluated against RLAR, AGEs, and DPPH. Consequently, we conclude that SHL extract and its constituents contribute at least in part in RLAR and AGEs inhibition. Furthermore, our results suggest SHL extract and its constituents as potential natural drugs to treat hyperglycemia-induced cataract and various diabetic complications.

## Figures and Tables

**Table 1:** Inhibitory effect of crude extract and fractions of *Stauntonia hexaphylla* leaves on rat lens aldose reductase (RLAR), advanced glycation end products (AGEs), and antioxidant (DPPH).

Extract and fractions		IC_50_ (*μ*g/mL)^a^		
		RLAR	AGEs	DPPH
Extract	70% EtOH	34.52 ± 2.21	422.51 ± 36.77	193.19 ± 19.01

Fractions	Hex	31.24 ± 2.24	270.51 ± 26.77	>2000
	CH_2_Cl_2_	24.87 + 0.98	222.53 ± 11.25	420.28 ± 41.23
	EtOAc	6.90 ± 0.34	50.07 ± 4.21	63.00 ± 6.20
	*n*-BuOH	14.98 ± 1.01	100.90 ± 9.32	254.84 ± 21.52
	Water	>50	>500	1109.59 ± 98.17

Positive controls	Quercetin^b^	5.48 ± 0.44	—	—
	Aminoguanidine^c^	—	90.70 ± 8.71	—
	L-ascorbic acid^d^	—	—	9.58 ± 7.89

^a^The IC_50_ value was defined as a mean ± SEM of half-maximal inhibitory concentration from three independent experiments performed in duplicate.

^b^Quercetin is the positive control for aldose reductase.

^c^Aminoguanidine is the positive control for advanced glycation end products.

^d^L-ascorbic acid is the positive control for DPPH.

**Table 2:** Inhibitory effect of compounds isolated from *Stauntonia hexaphylla* leaves on rat lens aldose reductase (RLAR).

Compounds	IC_50_ (*μ*M)^a^
3-(3,4-Dihydroxyphenyl)-propionic acid (**1**)	>1000
Chlorogenic acid (**2**)	8.35 ± 0.45
Neochlorogenic acid (**3**)	72.03 ± 25.77
Calceolarioside B (**4**)	23.99 ± 2.30
Luteolin-3′-*O*-*β*-D-glucopyranoside (**5**)	7.34 ± 0.35
Quercetin-3-*O*-*β*-D-glucopyranoside (**6**)	10.40 ± 1.38
Luteolin-7-*O*-*β*-D-glucopyranoside (**7**)	16.10 ± 1.20

Quercetin^b^	18.09 ± 1.30

^a^The IC_50_ value was defined as a mean ± SEM of half-maximal inhibitory concentration from three independent experiments performed in duplicate.

^b^Quercetin is the positive control for aldose reductase.

**Table 3:** Inhibitory effect of compounds isolated from *Stauntonia hexaphylla* leaves on advanced glycation end products (AGEs) formation.

Compounds	IC_50_ (*μ*M)^a^
3-(3,4-Dihydroxyphenyl)-propionic acid (**1**)	184.06 ± 17.62
Chlorogenic acid (**2**)	>1000
Neochlorogenic acid (**3**)	115.07 ± 10.47
Calceolarioside B (**4**)	>1000
Luteolin-3′-*O*-*β*-D-glucopyranoside (**5**)	117.80 ± 11.46
Quercetin-3-*O*-*β*-D-glucopyranoside (**6**)	>1000
Luteolin-7-*O*-*β*-D-glucopyranoside (**7**)	>1000

Aminoguanidine^b^	820.44 ± 79.42

^a^The IC_50_ value was defined as a mean ± SEM of half-maximal inhibitory concentration from three independent experiments performed in duplicate.

^b^Aminoguanidine is the positive control for advanced glycation end products.

**Table 4:** Inhibitory effect of compounds isolated from *Stauntonia hexaphylla* leaves on DPPH radical scavenging activity.

Compounds	IC_50_ (*μ*M)^a^
3-(3,4-Dihydroxyphenyl)-propionic acid (**1**)	109.73 ± 9.39
Chlorogenic acid (**2**)	162.60 ± 14.39
Neochlorogenic acid (**3**)	>1000
Calceolarioside B (**4**)	94.60 ± 6.86
Luteolin-3′-*O*-*β*-D-glucopyranoside (**5**)	96.28 ± 9.19
Quercetin-3-*O*-*β*-D-glucopyranoside (**6**)	122.34 ± 10.94
Luteolin-7-*O*-*β*-D-glucopyranoside (**7**)	>1000

L-ascorbic acid^b^	54.39 ± 5.73

^a^The IC_50_ value was defined as a mean ± SEM of half-maximal inhibitory concentration from three independent experiments performed in duplicate.

^b^L-ascorbic acid is the positive control for DPPH radical scavenging activity.
